# Supplementary material for: The Role of IL-9 Polymorphisms and Serum IL-9 Levels in Carcinogenesis and Survival Rate for Laryngeal Squamous Cell Carcinoma
Source: Cells. 2021 Mar 9;10(3):601. doi: 10.3390/cells10030601 (PMC8001846; doi:10.3390/cells10030601)
Supplement: Supplementary file 1 [file cells-10-00601-s001.pdf]

SUPPLEMENTARY MATERIAL

Table S1. Binomial logistic regression analysis of *IL-9:* rs1859430, rs2069870, rs11741137, rs2069885, rs2069884 in healthy control and patients with LSCC groups.

Model	Genotype/allele	OR[1] (95% CI[2])	*p*- Value[3]	AIC[4]
		***IL-9* rs1859430**		
Codominant	G/A *vs.* A/A	0.889 (0.655-1.208)	0.452	1092.101
	G/G *vs.* A/A	1.040 (0.554-1.950)	0.903	
Dominant	G/A+G/G *vs.* A/A	0.909 (0.679-1.217)	0.522	1090.321
Recessive	G/G *vs.* A/A+G/A	1.083 (0.583-2.014)	0.800	1090.669
Overdominant	G/A *vs.* A/A+G/G	0.886 (0.655-1.199)	0.433	1090.116
Additive	G	0.949 (0.748-1.205)	0.669	1090.549
		***IL-9* rs2069870**		
Codominant	A/G *vs.* G/G	0.892 (0.665-1.195)	0.443	1092.141
	A/A *vs.* G/G			
Dominant	A/G+A/A *vs.* G/G	0.892 (0.665-1.195)	0.443	1090.141
Recessive	A/A *vs.* G/G+A/G	-	-	-
Overdominant	A/G *vs.* G/G+A/A	0.892 (0.665-1.195)	0.443	1090.141
Additive	A	0.892 (0.665-1.195)	0.443	1090.141
		***IL-9* rs11741137**		
Codominant	C/T *vs.* C/C	0.894 (0.651-1.228)	0.490	1091.826
	T/T *vs.* C/C	1.300 (0.539-3.137)	0.559	
Dominant	C/T+T/T *vs.* C/C	0.923 (0.679-1.255)	0.609	1090.471
Recessive	T/T *vs.* C/C+C/T	1.343 (0.559-3.225)	0.510	1090.305
Overdominant	C/T *vs.* C/C+T/T	0.886 (0.646-1.215)	0.452	1090.163
Additive	T	0.965 (0.736-1.266)	0.800	1090.668
		***IL-9* rs2069885**		
Codominant	G/A *vs.* A/A	0.867 (0.629-1.194)	0.383	1091.268
	G/G *vs.* A/A	1.410 (0.575-3.457)	0.453	
Dominant	G/A+G/G *vs.* A/A	0.903 (0.663-1.231)	0.518	1090.314
Recessive	G/G *vs.* A/A+G/A	1.468 (0.601-3.583)	0.399	1090.035
Overdominant	G/A *vs.* A/A+G/G	0.857 (0.623-1.178)	0.342	1089.822
Additive	G	0.956 (0.727-1.257)	0.747	1090.628
		***IL-9* rs2069884**		
Codominant	T/G *vs.* G/G	0.867 (0.629-1.194)	0.383	1091.268
	T/T *vs.* G/G	1.410 (0.575-3.457)	0.453	
Dominant	T/G+T/T vs. G/G	0.903 (0.663-1.231)	0.518	1090.314
Recessive	T/T *vs.* G/G+T/G	1.468 (0.601-3.583)	0.399	1090.035
Overdominant	T/G *vs.* G/G+T/T	0.857 (0.623-1.178)	0.342	1089.822
Additive	T	0.956 (0.727-1.257)	0.747	1090.628

[1]OR: odds ratio; [2]CI: confidence interval; [3]*p*-Value: significance level $p<0.05$; [4]AIC: Akaike information criterion.

Table S2. Frequencies of *IL-9*: rs1859430, rs2069870, rs11741137, rs2069885, and rs2069884 in the control group and patients with LSCC early stage and advanced stage subgroups.

Gene	Genotype / Allele	Control group n (%) (n=533)	Early-stage of LSCC[1] subgroup (I + II) (%), n=169	*p*-Value	Advanced stage (III + IV) (proc.), n=131	*p*-Value	*p*-Value[2]*
IL-9 rs1859430	**G/G**	322 (60.4)	113 (66.9)	0.168	75 (57.3)	0.701	
	**A/G**	183 (34.3)	45 (26.6)		50 (38.2)		
	**A/A**	28 (5.3)	11 (6.5)		6 (4.6)		
	**Total**	533 (100)	169 (100)		131 (100)		
	**Allele**						
	**G**	827 (77.6)	271 (80.2)		200 (76.3)		
	**A**	239 (22.4)	67 (19.8)		62 (23.7)		
IL-9 rs2069870	**A/A**	325 (61.0)	113 (66.9)	0.169	78 (59.5)	0.763	
	**A/G**	208 (39.0)	56 (33.1)		53 (40.5)		
	**G/G**	-	-		-		
	**Total**	533 (100)	169 (100)		131 (100)		
	**Allele**						
	**A**	858 (80.5)	282 (83.4)		209 (79.8)		
	**G**	208 (19.5)	56 (16.6)		53 (20.2)		
IL-9 rs11741137	**C/C**	364 (68.3)	123 (72.8)	0.253	87 (66.4)	0.916	
	**C/T**	157 (29.5)	40 (23.7)		41 (31.3)		
	**T/T**	12 (2.3)	6 (3.6)		3 (2.3)		
	**Total**	533 (100)	169 (100)		131 (100)		
	**Allele**						
	**C**	885 (83.0)	286 (84.6)		215 (82.1)		
	**T**	181(17.)	52 (15.4)		47 (17.9)		
IL-9 rs2069885	**G/G**	367 (68.9)	123 (72.8)	0.246	90 (68.7)	0.987	
	**G/A**	155 (29.1)	40 (23.7)		38 (29.0)		
	**A/A**	11 (2.1)	6 (3.6)		3 (2.3)		
	**Total**	533 (100)	169 (100)		131 (100)		
	**Allele**						
	**G**	889 (83.4)	286 (84.6)		218 (83.2)		
	**A**	177 (16.6)	52 (15.4)		44 (16.8)		
IL-9 rs2069884	**G/G**	367 (68.9)	123 (72.8)	0.246	90 (68.7)	0.987	
	**G/T**	155 (29.1)	40 (23.7)		38 (29.0)		
	**T/T**	11 (2.1)	6 (3.6)		3 (2.3)		
	**Total**	533 (100)	169 (100)		131 (100)		
	**Allele**						
	**G**	889 (83.4)	286 (84.6)		218 (83.2)		
	**T**	177 (16.6)	52 (15.4)		44 (16.8)		

[1]LSCC: Laryngeal squamous cell carcinoma; [2]*p*-Value: significance level $p<0.05$; *Early stage *vs*. advanced stage.

Table 3. Frequencies of *IL-9* rs1859430, rs2069870, rs11741137, rs2069885, and rs2069884 genotypes and alleles in the control group and LSCC patients with no metastasis and with metastasis to the neck lymph nodes subgroups.

Gene	Genotype / Allele	Control group (proc.), n=533	No metastasis to the neck lymph nodes (%), n=242	*p*-Value[1]	Metastasis to the neck lymph nodes (%), n=58	*p*-Value[1]	*p*-Value[1,*]
IL-9 rs1859430	**G/G**	322 (60.4)	157 (64.9)	0.487	31 (53.4)	0.431	

	**A/G**	183 (34.3)	73 (30.2)		22 (37.9)	
	**A/A**	28 (5.3)	12 (5.0)		5 (8.6)	
	**Total**	533 (100)	242 (100)		58 (100)	
	**Allele**					
	**G**	827 (77.6)	387 (80.0)		84 (72.4)	
	**A**	239 (22.4)	97 (20.)		32 (27.6)	
IL-9 rs2069870	**A/A**	325 (61.0)	155 (64.0)	0.414	36 (62.1)	0.871
	**A/G**	208 (39.0)	87 (36.0)		22 (37.9)	
	**G/G**	-	-		-	
	**Total**	533 (100)	242 (100)		58 (100)	
	**Allele**					
	**A**	858 (80.5)	397 (82.0)		94 (81.0)	
	**G**	208 (19.5)	87 (18.0)		21 (19.0)	
IL-9 rs11741137	**C/C**	364 (68.3)	172 (71.1)	0.562	38 (65.5)	0.811
	**C/T**	157 (29.5)	63 (26.0)		18 (31.0)	
	**T/T**	12 (2.3)	7 (2.9)		2 (3.4)	
	**Total**	533 (100)	242 (100)		58 (100)	
	**Allele**					
	**C**	885 (83.0)	407 (84.1)		94 (81.0)	
	**T**	181 (17.0)	77(15.9)		22 (19.0)	
IL-9 rs2069885	**G/G**	367 (68.9)	173 (71.5)	0.504	40 (69.0)	0.780
	**G/A**	155 (29.1)	62 (25.6)		16 (27.6)	
	**A/A**	11 (2.1)	7 (2.9)		2 (3.4)	
	**Total**	533 (100)	242 (100)		58 (100)	
	**Allele**					
	**G**	889 (83.4)	408 (84.3)		96 (82.8)	
	**A**	177 (16.6)	76 (15.7)		20 (17.2)	
IL-9 rs2069884	**G/G**	367 (68.9)	173 (71.5)	0.504	40 (69.0)	0.780
	**G/T**	155 (29.1)	62 (25.6)		16 (27.6)	
	**T/T**	11 (2.1)	7 (2.9)		2 (3.4)	
	**Total**	533 (100)	242 (100)		58 (100)	
	**Allele**					
	**G**	889 (83.4)	408 (84.3)		96 (82.8)	
	**T**	177 (16.6)	76 (15.7)		20 (17.2)	

[1]*p*-Value: significance level $p<0.05$; *No metastasis to neck lymph nodes *vs*. metastasis to neck lymph nodes.
